# Editorial on Special Issue: “Smart Polymer Hydrogels: Synthesis, Properties and Applications—Volume I”

**DOI:** 10.3390/gels9020084

**Published:** 2023-01-19

**Authors:** Wei Ji

**Affiliations:** Key Laboratory of Biorheological Science and Technology, Ministry of Education, College of Bioengineering, Chongqing University, Chongqing 400044, China; weiji@cqu.edu.cn

Smart polymer hydrogels are soft materials formed by crosslinking with various covalent and non-covalent interactions. They can respond to different chemical and physical external stimuli, such as pH, temperature, light, redox agents, electric or magnetic field, and so on. Due to the advantages of biocompatibility, stimuli responsiveness and low cost, smart polymer hydrogels have gained increasing interest as promising soft materials for their tremendous potential applications in biomedical and nanotechnological fields. Fortunately, in this Special Issue, we obtained 11 papers from researchers working in the hydrogel related fields. 

The papers in this Special Issue present the research results on the synthesis, properties and applications of smart polymer hydrogels. To obtain a new biohybrid gel structure, Sandu et al. present a strategy by coupling synthetic polymers with natural compounds using a spiroacetal polymer and alginate at different ratios. The release of carvacrol, an encapsulated bioactive compound, could be controlled by changing the ratio of the synthetic polymer [1]. Mrohs et al. reported the effect of multivalent allylammonium-based crosslinkers on the synthesis of homogeneous, highly swelling diallyldimethylammonium chloride hydrogels, which indicated that homogeneity plays important role for the formation of coherent gels with low cross-linking densities [2]. To evaluate the physical stability of starch-based hydrogels via high-pressure processing, Larrea-Wachtendorff et al. performed the accelerated methods based on temperature sweep tests and oscillatory rheological measurements, as well as temperature cycling tests, which could be used in predicting the physical stability of starch-based hydrogels [3].

As promising drug delivery and tissue engineering scaffolds, hydrogels have attracted great interest from researchers for the potential applications in tumour and disease treatment. Suhail et al. reported novel pH-responsive polymeric β-cyclodextrin-based hydrogels using the free radical polymerization technique, which could be utilized for the controlled delivery of theophylline [4]. Mahmood et al. synthesized *Linum usitatissimum* mucilage polymer-based hydrogel for sustained release of oral drugs. Compared to the conventional synthetic polymers, the developed above hydrogel showed many-fold benefits in the in vitro experiments for oral delivery of drugs [5]. Harui et al. reported a hyaluronidase and anti-CTLA-4 containing hydrogel to target tumour draining lymph nodes, which further improved the anti-tumour efficacy examined by employing live and ex vivo imaging [6]. Lee et al. reported a hydrogel-based fully realized and passive implantable valve for the treatment of hydrocephalic fluid retention. The reproducibility of the hydrogel-based valve and sensor functions was demonstrated, indicating the system’s potential applications as a chronic implant [7]. Ye et al. summarized the recent advances of hydrogels as soft materials in the tissue engineering area for the treatment of oral disease [8].

Hydrogels have three-dimensional pore networks showing the application in cleaning the wastewater; Hamri et al. reported a theoretical model based on docking simulations and applied the model to analyze the adsorption process of a polymer-based hydrogel in cleaning of wastewater [9]. Stagno et al. presented a method for non-invasive assessment of a PVA hydrogel in removing metal corrosion products on stones by portable Nuclear Magnetic Resonance (NMR), indicating that the NMR protocol cold be used for in situ analysis of cleaning efficacy and the effect of different hydrogels on different materials [10]. Xu et al. synthesized a new polymer hydrogel in the presence of spiropyran dimethacrylate mechanophore crosslinker. The developed polymer hydrogel could display the sole mechanochromic behavior, holding great potential in outdoor strain sensors [11].

We hope that the papers published in this Special Issue will inspire the design and synthesis of more smart polymer hydrogels for biomedical and nanotechnological applications. The Editorial Team thanks all the authors who contributed to the Special Issue of “Smart Polymer Hydrogels: Synthesis, Properties and Applications—Volume I”. 
